# Gastric cancer mesenchymal stem cells derived IL-8 induces PD-L1 expression in gastric cancer cells via STAT3/mTOR-c-Myc signal axis

**DOI:** 10.1038/s41419-018-0988-9

**Published:** 2018-09-11

**Authors:** Li Sun, Qianqian Wang, Bin Chen, Yuanyuan Zhao, Bo Shen, Hua Wang, Jing Xu, Miaolin Zhu, Xiangdong Zhao, Changgen Xu, Zhihong Chen, Mei Wang, Wenrong Xu, Wei Zhu

**Affiliations:** 1 School of Medicine, Jiangsu University, Zhenjiang, Jiangsu China; 2 Department of oncology, Jiangsu Cancer Hospital Affiliated to Nanjing Medical University, Nanjing, Jiangsu China; 3 Affiliated Hospital of Jiangsu University, Zhenjiang, Jiangsu China; 4 Zhenjiang Provincial Blood Center, Zhenjiang, Jiangsu China; 5 Department of Gastrointestinal Surgery, Affiliated People’s Hospital of Jiangsu University, Zhenjiang, Jiangsu China

## Abstract

The expression of PD-L1 in tumor cells is one of the main causes of tumor immune escape. However, the exact mechanism for regulating PD-L1 expression in gastric cancer (GC) cells remains unclear. Our previous studies have shown that mesenchymal stem cells (MSCs) exert broad immunosuppressive potential, modulating the activity of cells either in innate or adaptive immune system to promote tumor progress. This study aims to investigate whether GCMSCs regulate the PD-L1 expression in GC cells and explore the specific molecular mechanism. The results have shown that GCMSCs enhanced PD-L1 expression in GC cells resulting in the resistance of GC cells to CD8^+^ T cells cytotoxicity. However, this resistance was attenuated with IL-8 inhibition. Further studies proved that IL-8 derived from GCMSCs induced PD-L1 expression in GC cells via c-Myc regulated by STAT3 and mTOR signaling pathways. Our data indicated that blocking IL-8 derived from GCMSCs may overcome the immune escape induced by PD-L1 in GC cells and provide a potential strategy to enhance the immunotherapy efficiency in GC.

## Introduction

Gastric cancer (GC) is the fourth most common malignant tumor, and the second leading cause of cancer-related death worldwide^1^. Although remarkable achievements in surgical and other therapies have been obtained, there is still a poor 5-year survival rate among GC patients^2^. In recent years, immunotherapy is a major breakthrough in cancer therapy and clinical trials with PD-1/PD-L1 antibodies have shown unprecedented responses in GC.

PD-L1 has been reported to be overexpressed in several malignant tumors and the mechanisms of PD-L1 regulation was multifaceted, such as genomic aberrations, mRNA stability, transcriptional control, protein stability and oncogenic signaling^3,4^. Furthermore, PD-L1 expression was associated with the resistance to anticancer therapies and the poor prognosis^5–7^. Takahashi et al. reported that in metastatic GC patients, high serum levels of sPD-L1 were correlated with worse overall survival on the first-line chemotherapy^8^. Böger et al. found that PD-L1 expression was not only an independent survival prognosticator but also correlated with distinct clinico-pathological patient characteristics^9^. C-Myc, serving as a well-known oncogene, is thought to be involved in tumor initiation and development. Casey et al. proved that Myc bound directly to the promoters of the PD-L1 genes in mouse T cell acute lymphoblastic leukemia^10^. Sato et al. reported that STAT3 was required for PD-L1 up-regulation in prostate cancer cell lines and osteosarcoma cell lines^11^. AKT/mTOR signaling pathway represents a convergence point for many oncogenes activation and is also associated with PD-L1 regulation in non-small cell lung cancer^12^. It has been reported that INF-γ played a vital role in PD-L1 induction in many cancers and then caused their resistance to NK cells^13,14^. Mimura et al. also found that PD-L1 expression could be regulated by INF-γ in GC^15^. However, the specific molecular mechanism for regulating PD-L1 expression in GC cells remains unclear.

Mesenchymal stem cells (MSCs) with multiple differentiation potential and immune modulating function, are one of the most important cell components of tumor microenvironment (TME)^16^. Our previous studies proved that bone marrow MSCs (BMMSCs) served as primary cellular components contribute to the tumor progress and mainly via secretory cytokines or exosomes^17–19^. Further, we isolated GCMSCs from GC tissues which had a stronger tumor promoting effect compared with BMMSCs. Kim et al. also found that GCMSCs contributed to the formation and progress of GC^20^. In addition, we found that GCMSCs exert broad immunosuppressive potential, which increased the proportion of regulatory T cells and decreased that of Th17 cells in peripheral blood mononuclear cells (PBMCs)^21^. However, the exact mechanism still remains unclear.

This study aims to investigate whether GCMSCs regulate the PD-L1 expression in GC cells and explore the specific molecular mechanism. The results have shown that IL-8 derived from GCMSCs induced PD-L1 expression in GC cells via c-Myc regulated by STAT3 and mTOR signaling pathways. Moreover, IL-8 inhibition weakened GCMSCs protective effects on GC cells against CD8^+^ T cells cytotoxicity. In brief, our data indicated that inhibition of IL-8 derived from GCMSCs may overcome the immune escape induced by PD-L1 in GC cells and provide a potential strategy to enhance the efficacy of PD-L1 antibody immunotherapy in GC.

## Materials and methods

### MSCs, cell lines, and cell-culture

GC tissues were obtained from GC patients of Jiangsu Cancer Hospital Affiliated to Nanjing Medical University and the Affiliated People’s Hospital of Jiangsu University. The procedure was approved by the Ethics Committee of Jiangsu University. The informed consents were obtained from all subjects. GCMSCs were isolated from human GC tissues as previously described^22^. In brief, fresh tissues were cut into about 1 mm^3^-sized pieces and adhered to 35 mm cell culture dishes (Corning, USA) and were cultured in Dulbecco’s Modified Eagle Medium (DMEM, Gibco, USA) with 10% fetal bovine serum (FBS, Gibco) at 37 °C in humid air with 5% CO_2_. The culture medium was refreshed every three days. When the confluence of fibroblast-like cells reached about 80%, the cells were further expanded up to five passages and used for the subsequent experiments.

BMMSCs were isolated from healthy donors and this procedure was approved by the Ethics Committee of Jiangsu University. The BM cells were diluted with equal volume of phosphate-buffered saline (PBS) and separated over a gradient of 1.077 g/ml Ficoll (Dakewe, China), washed with PBS and cultured in DMEM with 10% FBS at 37 °C in humid air with 5% CO_2_. After about 14 days, the adherent cells were trypsinized and passaged up to five passages for use.

The human GC cell lines (MGC-803, SGC-7901, HGC-27 and BGC-823) were obtained from the Chinese Academy of Sciences Type Culture Collection Committee Cell Bank (Shanghai, China). MGC-803 and BGC-823 were cultured in DMEM with 10% FBS. SGC-7901 and HGC-27 were cultured in RPMI 1640 (Gibco) with 10% FBS at 37 °C in humid air with 5% CO_2_.

### Conditioned medium of BMMSCs (BMMSC-CM) and GCMSCs (GCMSC-CM) preparation

BMMSCs or GCMSCs were cultured in cell culture flasks and the culture medium was refreshed when the confluence reached about 70%. After 48 h, conditioned medium was harvested and centrifuged at 1000*g* for 5 min to remove cells, then filtered through a 0.22 μm membrane (Millipore, Germany) and stored at −80 °C until use.

### Western blot

Cells and tissues were lysed in RIPA buffer containing protease inhibitor cocktail (Vazyme). The protein concentration of cell lysates was quantified by a BCA Protein Assay kit (Cwbio). The primary antibodies were anti-PD-L1 (1:1000, CST), anti-c-Myc (1:1000, CST), anti-p-S6 (1:1000, CST), anti-S6 (1: 1 000, CST), anti-p-STAT3 (1:1000, CST), anti-STAT3 (1:1000, CST), anti-p-AKT (1:1000, CST), anti-AKT (1:1000, CST), and anti-GAPDH (1:2000, Cwbio). HRP-conjugated secondary antibody (1:2000, CST) and ECL reagent (Millipore) were used for detection of immunoreactive proteins captured by ImageQuant LAS 4000 detection system.

### Flow cytometry

PD-L1 staining used standard protocols with human monoclonal antibody: PE-anti-PD-L1 (BD Biosciences). Data were acquired by a flow cytometer (FACSCalibur, BD Biosciences) and flow imaging analysis (Flowsight, Merck Millipore).

### Immunohistochemistry

Tissues were first formalin-fixed and paraffin-embedded, then dewaxed in xylene, rehydrated with gradient ethanol and treated in citrate buffer for antigen retrieval. Then samples were stained with rabbit anti-human PD-L1 antibody (1:200, CST) at 4 °C overnight, followed by incubation in secondary biotinylated anti-rabbit antibody for 30 min at 37 °C, and finally visualized with DAB solution and counterstained with haematoxylin. Each stained sample was evaluated by three senior pathologists and five sights were selected typically.

### ELISA

The concentration of sPD-L1 in culture supernatants was measured by ELISA kit (R&D Systems, USA) according to the manufacturer’s instructions. The concentration of sPD-L1 was calculated based on standard curve provided with the kit.

### Immunofluorescence

Cells were incubated overnight at 4 °C with the anti-rabbit antibody PD-L1 (1:200, CST). Secondary antibodies (1:500, CST/Abcam) were applied for 1 h at 37 °C. Then cells were mounted with hoechst and images were captured with a structured illumination microscopy (Nikon, SIM).

### Human cytokine array

A cytokine array was performed in each conditioned medium after 24 h of culture. Human cytokine array panel (Raybiotech, USA) was used according to the manufacturer’s instructions.

### Cytotoxicity assay

Antigen-specific priming of CD8^+^ T cells was following the procedure described by Wölfl et al.^23^. Briefly, the subpopulation of naïve CD8^+^ T cells were purified from PBMCs from healthy donors using human naïve CD8^+^ T cells isolation kit (Miltenyi Biotec). Monocyte-derived dendritic cells were generated followed by stimulation with GC cell lysates and then co-incubated with naïve CD8^+^ T cells. Next, CD8^+^ T cells were separated and co-cultured with GC cells at the ratio of 5:1. PD-L1 neutralizing antibody was added to the culture system with a concentration of 2 μg/ml (eBioscience). After 24 h, CD8^+^ T cells and dead cells were discarded and the survival GC cells were stained with crystal violet, imaged and counted. Three experiments were independently performed.

### Statistical analysis

Data were shown as mean ± standard deviation (SD). One-way analysis of variance (ANOVA) was used to analyze the data and *P* < 0.05 is considered to be significant.

## Results

### GC tissues expressed higher PD-L1 than corresponding adjacent normal tissues

**Fig. 1:**
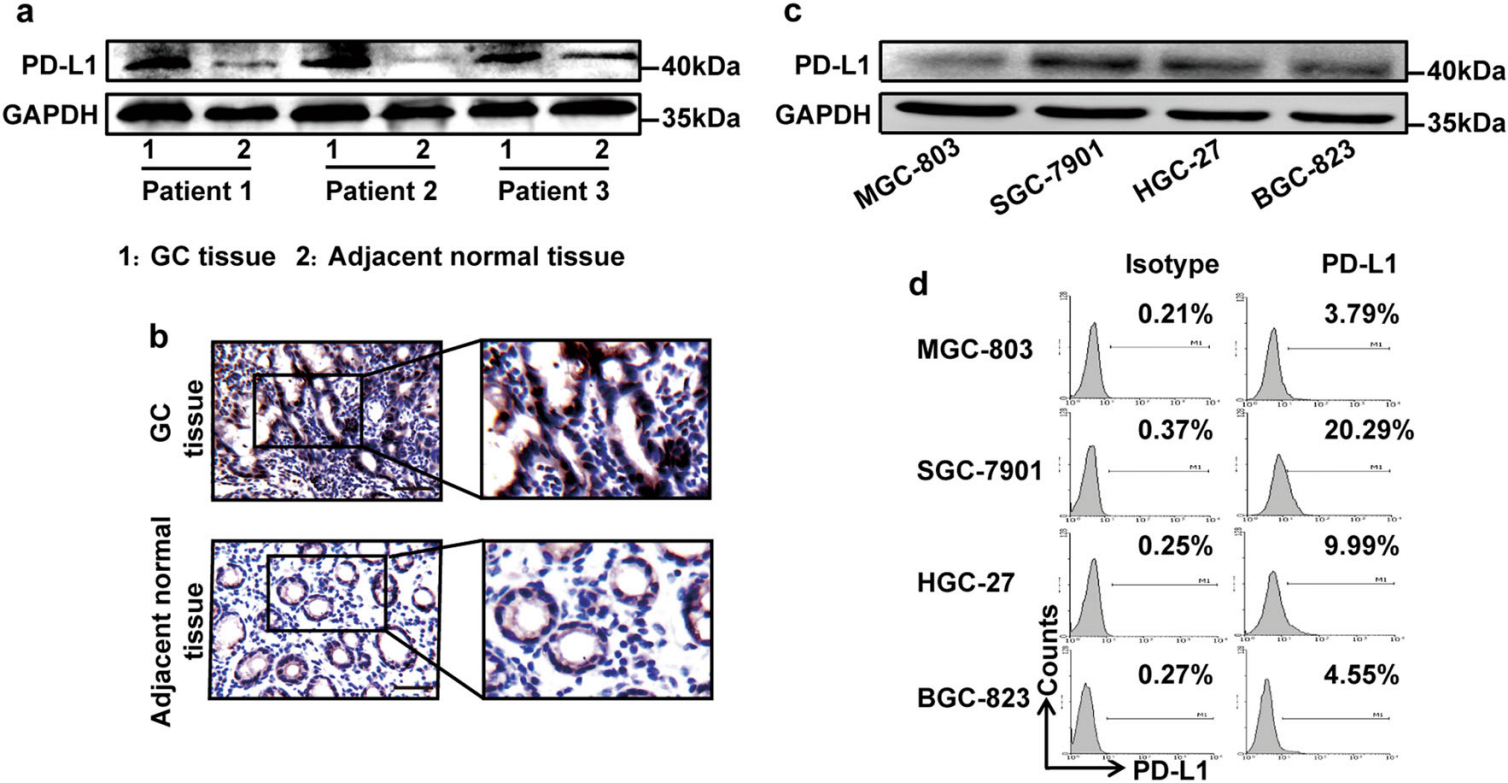
The expression of PD-L1 in GC tissues and GC cells. The PD-L1 expression of GC tissues and corresponding adjacent normal tissues was detected by western blot (**a**) and immunohistochemistry (**b**). Bar = 50 μm. The PD-L1 expression of GC cells was tested by western blot (**c**) and flow cytometry (**d**)

We chose PD-L1 positive GC tissues and detected the expression of PD-L1 in corresponding adjacent normal tissues. The results have shown that GC tissues expressed higher PD-L1 than corresponding adjacent normal tissues (Fig. 1a, b), which was consistent with previous literature reports. Further, we detected the expression of PD-L1 in several kinds of GC cells and found PD-L1 level was high in SGC-7901 and weak in MGC-803 (Fig. 1c, d). Therefore, these two GC cell lines were chosen for subsequent experiments.

### GCMSC-CM up-regulated the expression of PD-L1 in GC cells

**Fig. 2:**
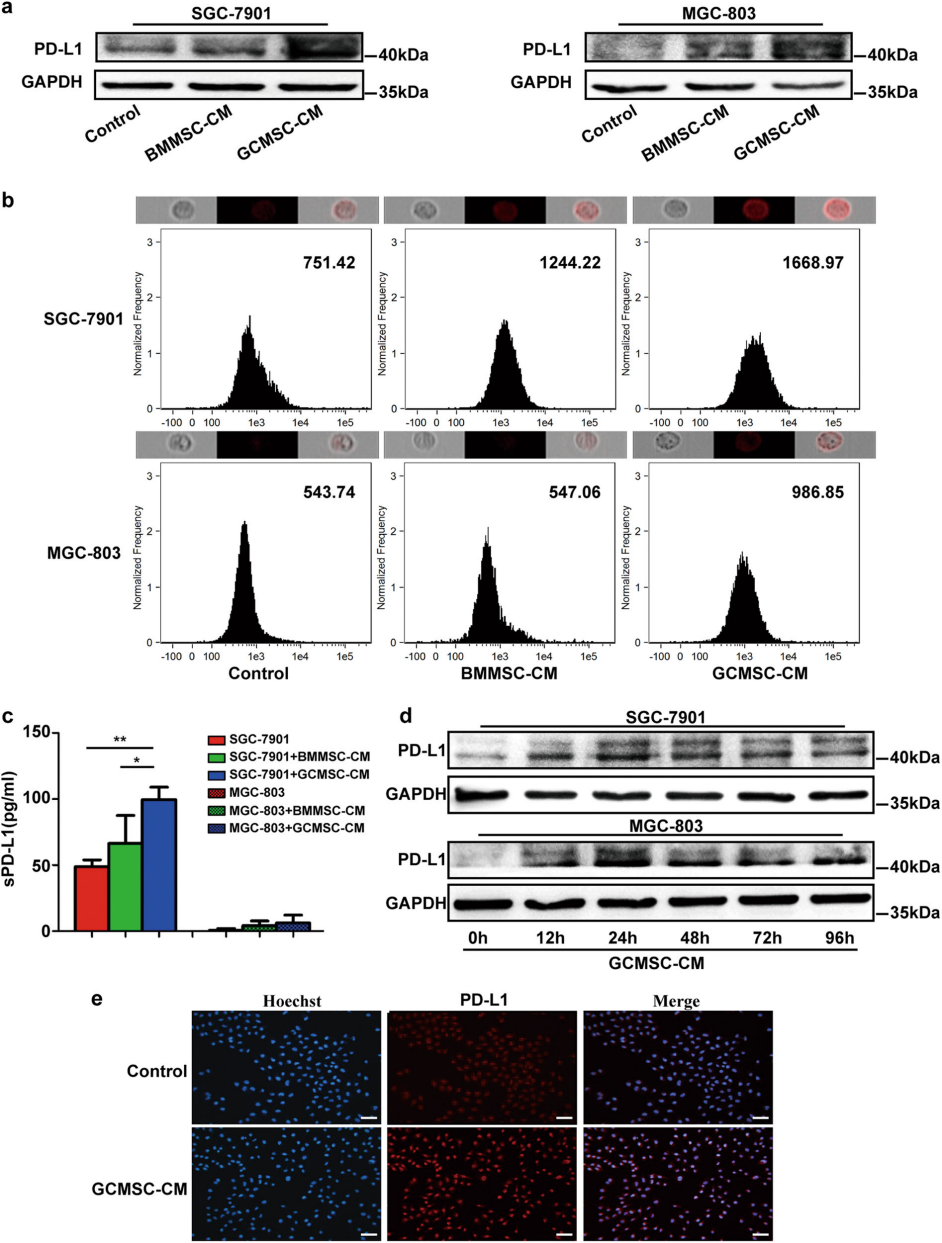
The expression of PD-L1 in GC cells treated with GCMSC-CM. The PD-L1 expression of SGC-7901 and MGC-803 treated with BMMSC-CM or GCMSC-CM for 24 h was detected by western blot (**a**) and flow cytometry (**b**) (median was shown). Cells expressing different levels of PD-L1 (red fluorescence) were shown above corresponding flow cytometry histograms. **c** The sPD-L1 levels in culture supernatants of SGC-7901 and MGC-803 treated with BMMSC-CM or GCMSC-CM for 24 h were detected by ELISA. **d** The PD-L1 expression of SGC-7901 and MGC-803 treated with GCMSC-CM for 12, 24, 48, 72 and 96 h was detected by western blot. **e** The PD-L1 level of MGC-803 treated with GCMSC-CM for 24 h was evaluated by immunofluorescence. Bar = 50 μm. Data in **c** represents the mean ± SD of three repeated experiments (*n* = 3). GCMSCs were isolated from three different GC patients. *P < 0.05, **P < 0.01

Differentiation assay in vitro showed that GCMSCs differentiated into adipocytes and osteocytes successfully (Figure S1a in supplementary data). Immunophenotype analysis displayed that GCMSCs were positive expression of CD105, CD90, and CD29, nevertheless, negative expression of CD45, CD34, and CD19 (Figure S1b in supplementary data). SGC-7901 and MGC-803 were treated with BMMSC-CM or GCMSC-CM. We found that compared with BMMSC-CM, GCMSC-CM highly up-regulated the expression of PD-L1 in SGC-7901 and MGC-803 (Fig. 2a, b). The results also indicated that GCMSC-CM from different GC patients enhanced PD-L1 expression to varying degrees in SGC-7901 or MGC-803 (Figure S2 in supplementary data). At the same time, compared with BMMSC-CM, SGC-7901 secreted higher concentration of soluble PD-L1 (sPD-L1) treated with GCMSC-CM (Fig. 2c). Next, we detected the PD-L1 levels in SGC-7901 and MGC-803 treated with GCMSC-CM for 12, 24, 48, 72, and 96 h and PD-L1 expression was at a high level from 24 h (Fig. 2d). Further, we detected the expression of PD-L1 in MGC-803 treated with GCMSC-CM in 24 h by Immunofluorescence. The results confirmed that compared with the corresponding control group, GCMSC-CM up-regulated PD-L1 level in GC cells (Fig. 2e).

### GCMSC-CM up-regulated PD-L1 expression in GC cells via c-Myc

**Fig. 3:**
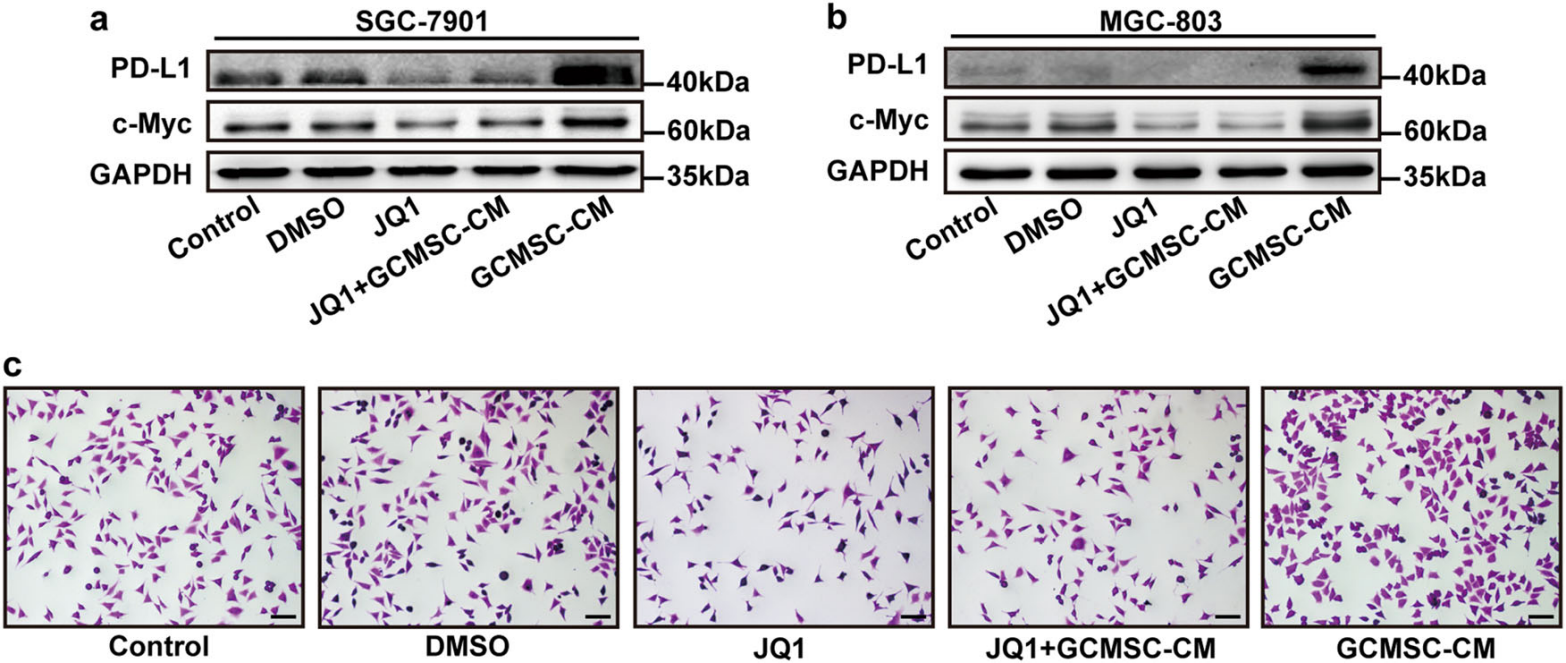
C-Myc played a role in GCMSC-CM-mediated PD-L1 up-regulation in GC cells. The expression of PD-L1 and c-Myc in SGC-7901 (**a**) and MGC-803 (**b**) treated with GCMSC-CM was detected by western blot. The concentration of JQ1 was 10 μM. **c** Cytotoxicity assay of MGC-803. MGC-803 was pre-treated with JQ1 and then treated with GCMSC-CM for 24 h. The survival cells were stained with crystal violet. Bar = 100 μm

Further, we investigated the mechanism of GCMSC-CM regulating PD-L1 expression in GC cells. The results have shown that GCMSC-CM could up-regulate c-Myc and PD-L1 expression in GC cells and the PD-L1 level decreased after JQ1, a BET domain inhibitor added, which inhibits BRD4 that acts as co-factors for Myc transcription (Fig. 3a, b). Next, MGC-803 was treated with GCMSC-CM for 24 h and then used for cytotoxicity assay. The number of survival cells in GCMSC-CM group was more than that in JQ1 + GCMSC-CM group (Fig. 3c).

### IL-8 derived from GCMSCs up-regulated PD-L1 expression in GC cells

**Fig. 4:**
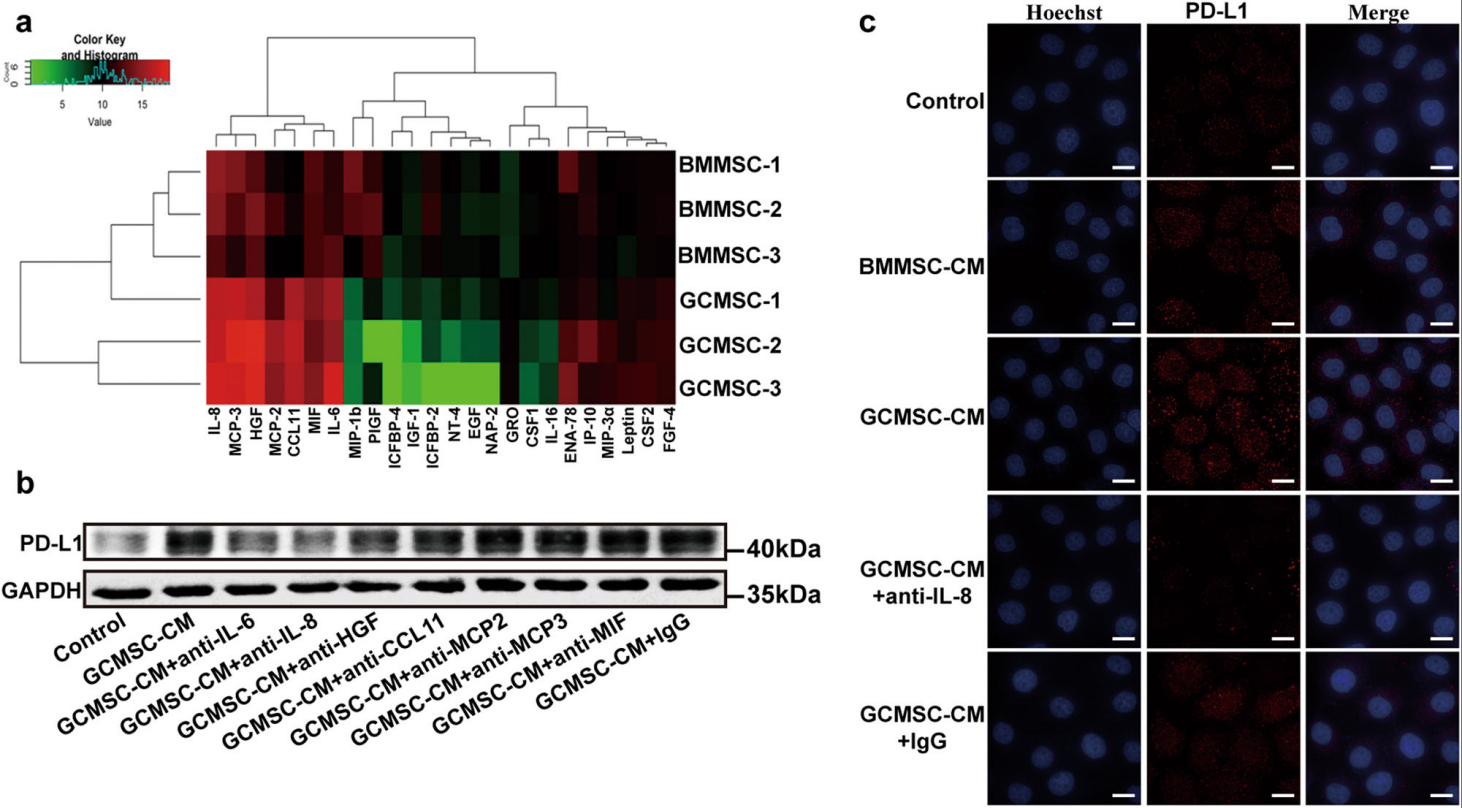
GCMSCs-derived IL-8 up-regulated PD-L1 expression in GC cells. **a** Cytokine array for screening different cytokines between BMMSC-CM and GCMSC-CM. **b** Each neutralizing antibody was added in GCMSC-CM and incubated at room temperature for 1 h and GCMSC-CM was then used to treat SGC-7901. **c** Immunofluorescence for the PD-L1 expression in SGC-7901 treated with GCMSC-CM. The concentration of IL-8 neutralizing antibody was 5 μg/ml (R&D Systems). Bar = 10 μm

To further elucidate GCMSCs contribute to PD-L1 expression mainly through secreting soluble cytokines, we compared the level of PD-L1 in SGC-7901 which was co-cultured with GCMSCs or treated with GCMSC-CM. The results have shown that the influence of GCMSCs and GCMSC-CM on PD-L1 up-regulation in SGC-7901 was similar (Figure S3 in supplementary data). For the reason that PD-L1 expression was reported to be controlled mainly by INF-γ in many tumors, we detected the secretion and expression of INF-γ by GCMSCs. We found that just like BMMSCs, the levels of GCMSCs secretion and expression of INF-γ were very low and there was no significant difference between them (Figure S4a and S4b in supplementary data). Then, the cytokine array was used to find the key factors in GCMSC-CM that regulated the expression of PD-L1 in GC cells. The results revealed that IL-6, IL-8, HGF, CCL11, MCP2, MCP3, and MIF were higher levels in GCMSC-CM than that in BMMSC-CM (Fig. 4a). In order to verify the factors that actually played a vital role in up-regulating PD-L1 expression, GCMSC-CM was used to treat SGC-7901 together with each cytokine neutralizing antibody. The results have shown that compared with GCMSC-CM group, the PD-L1 level dropped most obviously when IL-8 neutralizing antibody was added, followed by IL-6 neutralizing antibody (Fig. 4b). In addition, the level of IL-8 derived from GCMSCs was further detected by ELISA and the results have shown that the level of IL-8 secreted by GCMSCs was higher than that secreted by BMMSCs (Figure S4c in supplementary data). At the same time, we found that IL-8 neutralizing antibody weakened the PD-L1 expression in GCSC-CM group by Immunofluorescence. The results indicated that IL-8 played an important role in GCMSC-CM-mediated PD-L1 up-regulation in GC cells (Fig. 4c).

### GCMSCs-derived IL-8 induced PD-L1 expression in GC cells via c-Myc regulated by STAT3 and mTOR signaling pathways

**Fig. 5:**
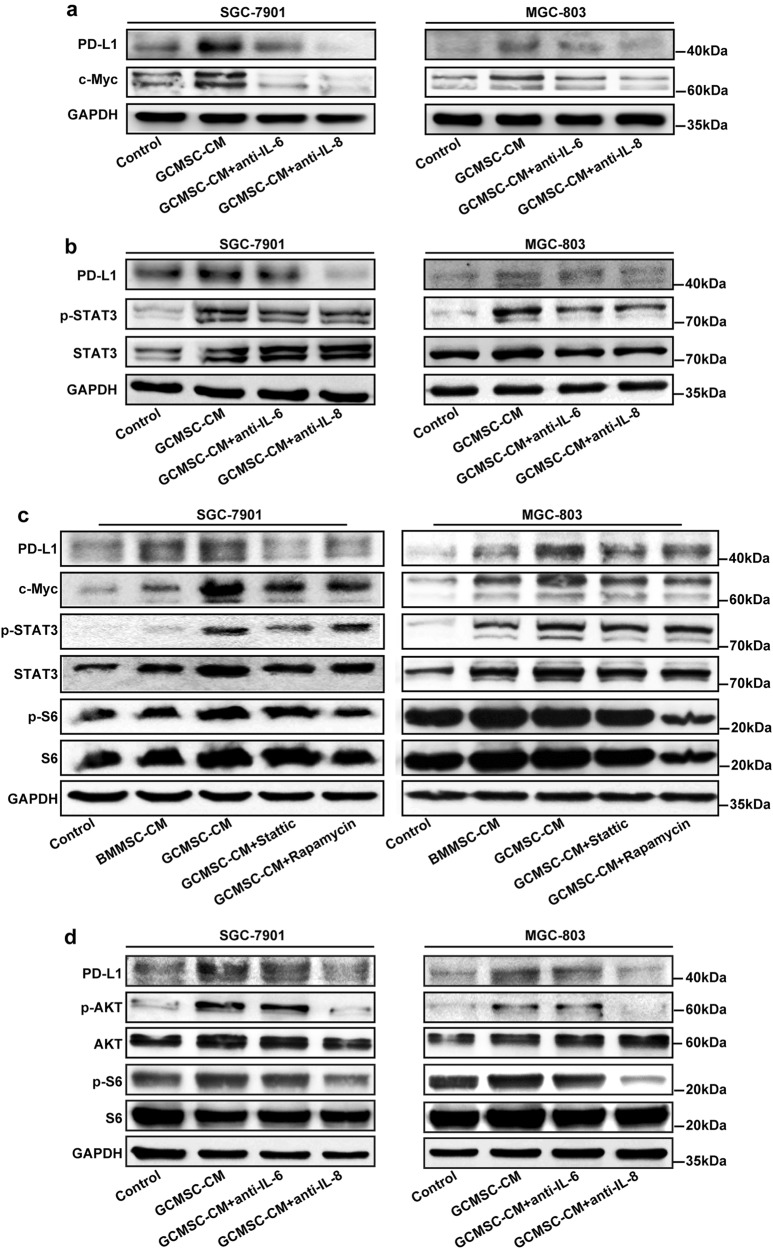
IL-8 derived from GCMSCs induced PD-L1 expression in GC cells via c-Myc regulated by STAT3 and mTOR signaling pathways. **a** The expression of PD-L1 and c-Myc in GC cells treated with GCMSC-CM was detected by western blot. **b** The phosphorylation and activation of STAT3 in GC cells was detected by western blot. **c** GC cells were treated with 5 μM of a STAT3 inhibitor (Stattic) or 0.1 μM of an mTOR inhibitor (Rapamycin). **d** The expression of PD-L1 and the phosphorylation of AKT and S6 ribosomal protein in GC cells was detected by western blot. The concentration of IL-6/IL-8 neutralizing antibody was 5 μg/ml

To investigate the exact molecular mechanism of IL-8 up-regulating PD-L1 level in GC cells, the expression of PD-L1 and c-Myc in GCMSC-CM group was tested with IL-6 and IL-8 neutralizing antibody added. As shown in Fig. 5a, compared with IL-6 inhibition, IL-8 inhibition weakened the up-regulation of PD-L1 and c-Myc more effectively. As described previously, STAT3 was involved in the regulation of PD-L1 and was a key signaling molecule downstream of IL-6^11,24^. This study showed that GCMSC-CM increased the phosphorylation and activation of STAT3, either IL-6 or IL-8 neutralizing antibody reversed this phenomenon (Fig. 5b). It indicated that both IL-6 and IL-8 could regulate PD-L1 via STAT3. AKT/mTOR signaling pathway also represents a convergence point for many oncogenes activation and associated with PD-L1 regulation and then we used STAT3 inhibitor, (Stattic) and mTOR inhibitor (Rapamycin) to further explore the mechanism of PD-L1 up-regulation which was induced by GCMSCs. The addition of Stattic significantly suppressed the phosphorylation and activation of STAT3 and inhibited the expression of PD-L1 and c-Myc in GC cells. At the same time, Rapamycin suppressed the expression of c-Myc and phosphorylation of S6 ribosomal protein, which was frequently used in determining the downstream activity of the mTOR complex, and also have an effect on PD-L1 (Fig. 5c). Therefore we speculated that besides STAT3, GCMSCs-derived IL-8 also could up-regulate PD-L1 in GC cells via AKT/mTOR signaling pathway. Then we detected the phosphorylation of AKT and S6 ribosomal protein in GC cells treated with GCMSC-CM with IL-6 or IL-8 neutralizing antibody added. The results have shown that the phosphorylation of AKT and S6 ribosomal protein in GC cells was suppressed in GCMSC-CM group with IL-8 neutralizing antibody added (Fig. 5d). To further clarify the regulatory effect of IL-8 on the expression of PD-L1 in GC cells, human recombinant IL-8 (rhIL-8) and rhIL-6 were used and the results have shown that compared with rhIL-6, rhIL-8 significantly increased the expression of PD-L1. Moreover, PD-L1 was up-regulated by rhIL-8 through STAT3/ mTOR-c-Myc signal axis in both SGC-7901 and MGC-803 (Figure S5 in supplementary data).

### GCMSCs-derived IL-8 protected GC cells against cytotoxic effect of CD8^+^ T cells

**Fig. 6:**
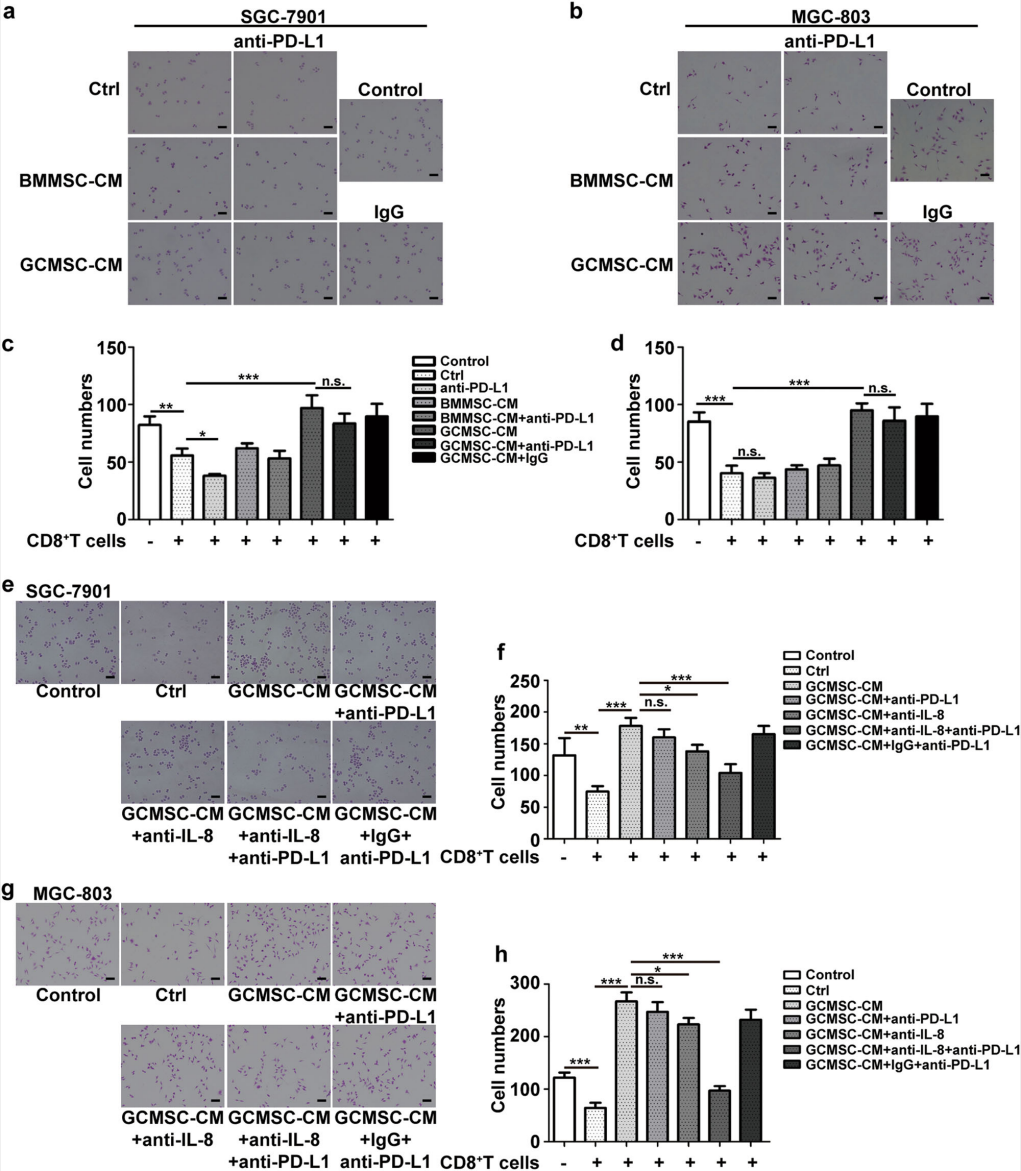
IL-8 in GCMSC-CM promoted GC cells resistance to cytotoxic effect of CD8^+^ T cells. **a, b** Cytotoxicity assay of GC cells. GC cells were treated with BMMSC-CM or GCMSC-CM for 24 h and then used for cytotoxicity assay. **c, d** Quantification of survival GC cell numbers. **e, g** Cytotoxicity assay of GC cells. IL-8 neutralizing antibody was added in GCMSC-CM and incubated at room temperature for 1 h and GCMSC-CM was then used to treat GC cells. After 24 h treatment, GC cells were used for cytotoxicity assay. **f, h** Quantification survival GC cell numbers. The concentration of PD-L1 neutralizing antibody was 2 μg/ml. Bar = 100 μm. Data in **c**, **d**, **f** and **h** represent the mean ± SD of three repeated experiments (*n* = 3). *P < 0.05, **P < 0.01, ***P < 0.001, n.s. not significant

In order to explore the effects of GCMSC-CM on the resistance of GC cells to CD8^+^ T cells, we did cytotoxicity assay. The results have shown that the survival cells in GCMSC-CM group were more than that in BMMSC-CM group and PD-L1 antibody had little effect on the survival cell number in GCMSC-CM group (Fig. 6a-d). Further, we found that compared with GCMSC-CM and GCMSC-CM + anti-PD-L1 groups, the survival cells were reduced in GCMSC-CM + anti-IL-8 + anti-PD-L1 group (Fig. 6e-h). These results above indicated that GCMSCs promotes GC cells against cytotoxic effect of CD8^+^ T cells through PD-L1 up-regulation in GC cells mediated by IL-8.

## Discussion

Several studies have reported that MSCs can promote the growth and metastasis of solid tumors directly^25^ or indirectly by inhibiting antitumor immune responses^26,27^. MSCs exert strong anti-inflammatory and immunosuppressive effects on immune cells through either cell–cell contact interactions or soluble factors they secreted, such as IDO, PGE2, and NO^28,29^. The key role of the resident stem cells in giving rise to the immunosuppressive TME was well reported^30^. For the reason that PD-L1 expression was reported to be controlled mainly by INF-γ in many tumors including GC^13–15^, we detected the secretion and expression of INF-γ by GCMSCs. We found that just like BMMSCs, the levels of GCMSCs secretion and expression of INF-γ were very low and there was no significant difference between them. Then, the cytokine array was used to find the key factors in GCMSC-CM that regulated the expression of PD-L1 in GC cells. The results revealed that the levels of IL-6, IL-8, HGF, CCL11, MCP2, MCP3, and MIF were higher in GCMSC-CM than that in BMMSC-CM. Cytokine neutralizing antibody further confirmed the effects of IL-6, IL-8, and HGF and among them, IL-8 increased PD-L1 expression in GC cells most obviously. Finally, we chose IL-8 as the key cytokine that up-regulated PD-L1 expression in GC cells mediated by GCMSC-CM. In future study, we will consider the combinative blockade of multiple cytokines to inhibit PD-L1 expression in GC cells.

PD-L1 has been reported to be overexpressed in several malignant tumors and was associated with the resistance to anticancer therapies and the poor prognosis^31,32^. The mechanisms of PD-L1 regulation was multifaceted and at highly complex manners^3,4^. As is well-known, JAK/STAT3 is a classical signaling pathway which plays a crucial part in the initiation and development of various cancers^24,33^. We found that the effects of IL-8 on PD-L1 expression was more remarkable than IL-6 even though they had similar influence on the activation of STAT3. Hence, we proposed that there was another mechanism regulated by IL-8. AKT/mTOR signaling pathway represents a convergence point for many oncogenes activation and also associated with PD-L1 regulation^12^, so we used Stattic (STAT3 inhibitor), and Rapamycin (mTOR inhibitor) to further determine the signaling pathways involved in GCMSC-CM-mediated up-regulation of PD-L1 expression in GC cellls.

C-Myc, serving as a well-known oncogene, is thought to be involved in tumor initiation and development. Casey et al. proved that Myc could regulate the antitumor immue response through PD-L1^10^. So we speculated that c-Myc plays a role in GCMSC-CM-mediated PD-L1 up-regulation in GC cells. We used JQ1, a novel small molecule that inhibits actions of BRDs and mainly down-regulates the expression of c-Myc to resist cells proliferation^34^. Therefore, we inhibited c-Myc expression by JQ1 and found that GCMSC-CM could up-regulate c-Myc and PD-L1. At the same time, the PD-L1 level decreased after the inhibition of c-Myc. So, we thought that c-Myc played an important role in GCMSC-CM-mediated PD-L1 up-regulation in GC cells.

Moreover, we tested the concentration of sPD-L1 in culture supernatants of SGC-7901 and MGC-803. The concentration of sPD-L1 in SGC-7901 GCMSC-CM group was higher than that in control or BMMSC-CM group. However, the concentration of sPD-L1 in culture supernatants of MGC-803 was still under the test line after GCMSC-CM treatment, probably because MGC-803 itself expressed very low PD-L1.

Cytotoxicity assay results have shown that the survival GC cell number in GCMSC-CM group was more than that in ctrl and BMMSC-CM groups, probably because GCMSC-CM protected GC cells against cytotoxic effect of CD8^+^ T cells. PD-L1 can deliver an inhibitory signal to PD-1 expressing CD8^+^ T cells, leading to suppression of the immune response by inducing functional exhaustion of CD8^+^ T cells. PD-L1 antibody was used to block the interaction between PD-L1 and PD-1 to improve cytotoxic effect of CD8^+^ T cells. However, PD-L1 antibody had little effect on the survival cell number in GCMSC-CM group and that might be because GCMSC-CM induced the up-regulation of PD-L1 in GC cells so that a certain dose of the antibody could not work and eventually, caused the immune escape of GC cells. Besides, compared with GCMSC-CM and GCMSC-CM + anti-PD-L1 groups, the survival cell number was decreased in GCMSC-CM + anti-IL-8 + anti-PD-L1 group. These results indicated that GCMSCs promotes GC cells against cytotoxic effect of CD8^+^ T cells through PD-L1 up-regulation in GC cells mediated by IL-8. This indicated that targeted inhibition of IL-8 may overcome PD-L1 antibody resistance in GC. In addition, with PD-L1 antibody added alone, SGC-7901 survival cells decreased slightly not the same as MGC-803 (no change), probably because MGC-803 expressed lower PD-L1 than SGC-7901.

**Fig. 7:**
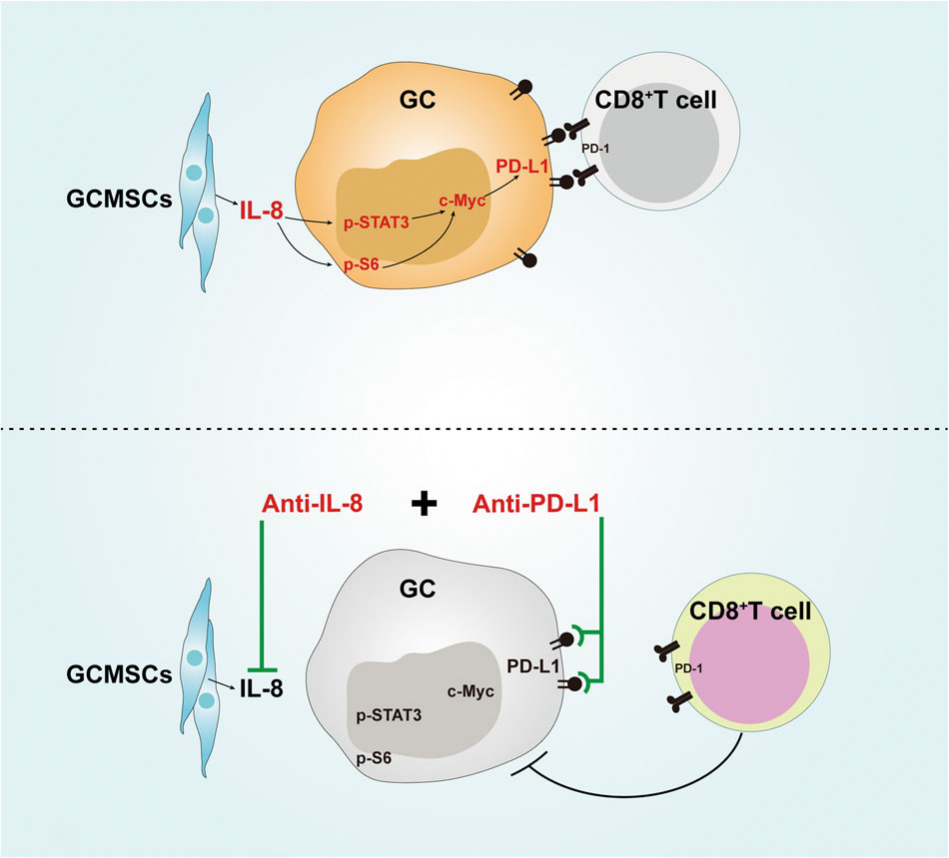
Schematic of proposed network of GCMSCs-mediated GC cells PD-L1 up-regulation and resistance to CD8^+^ T cells cytotoxicity. GCMSCs-derived IL-8 induced PD-L1 expression via c-Myc regulated by STAT3 and mTOR signaling pathways in GC cells. Combination with IL-8 inhibition enhanced the efficacy of PD-L1 antibody and promoted the cytotoxic effect of CD8^+^ T cells

In summary, this study have shown that GCMSC-CM enhanced PD-L1 expression in GC cells, and IL-8 played a key role in this process. GCMSCs-derived IL-8 induced PD-L1 expression in GC cells via c-Myc regulated by STAT3 and mTOR signaling pathways. In addition, IL-8 inhibition weakened GCMSCs protective effects on GC cells against CD8^+^ T cells cytotoxicity. Combination with IL-8 inhibition enhanced the efficacy of PD-L1 antibody and promoted the cytotoxic effect of CD8^+^ T cells (Fig. 7). Our data explains the mechanism of regulating PD-L1 expression in GC cells by GCMSCs and provide a potential strategy to enhance the efficacy of PD-L1 antibody immunotherapy in GC.

## Electronic supplementary material

Figure S1

Figure S2

Figure S3

Figure S4

Figure S5

supplementary figure legends
