# Supplementary figures and images for: Gastric cancer mesenchymal stem cells derived IL-8 induces PD-L1 expression in gastric cancer cells via STAT3/mTOR-c-Myc signal axis
Source: Cell Death Dis. 2018 Sep 11;9(9):928. doi: 10.1038/s41419-018-0988-9 (PMC6134105; doi:10.1038/s41419-018-0988-9)

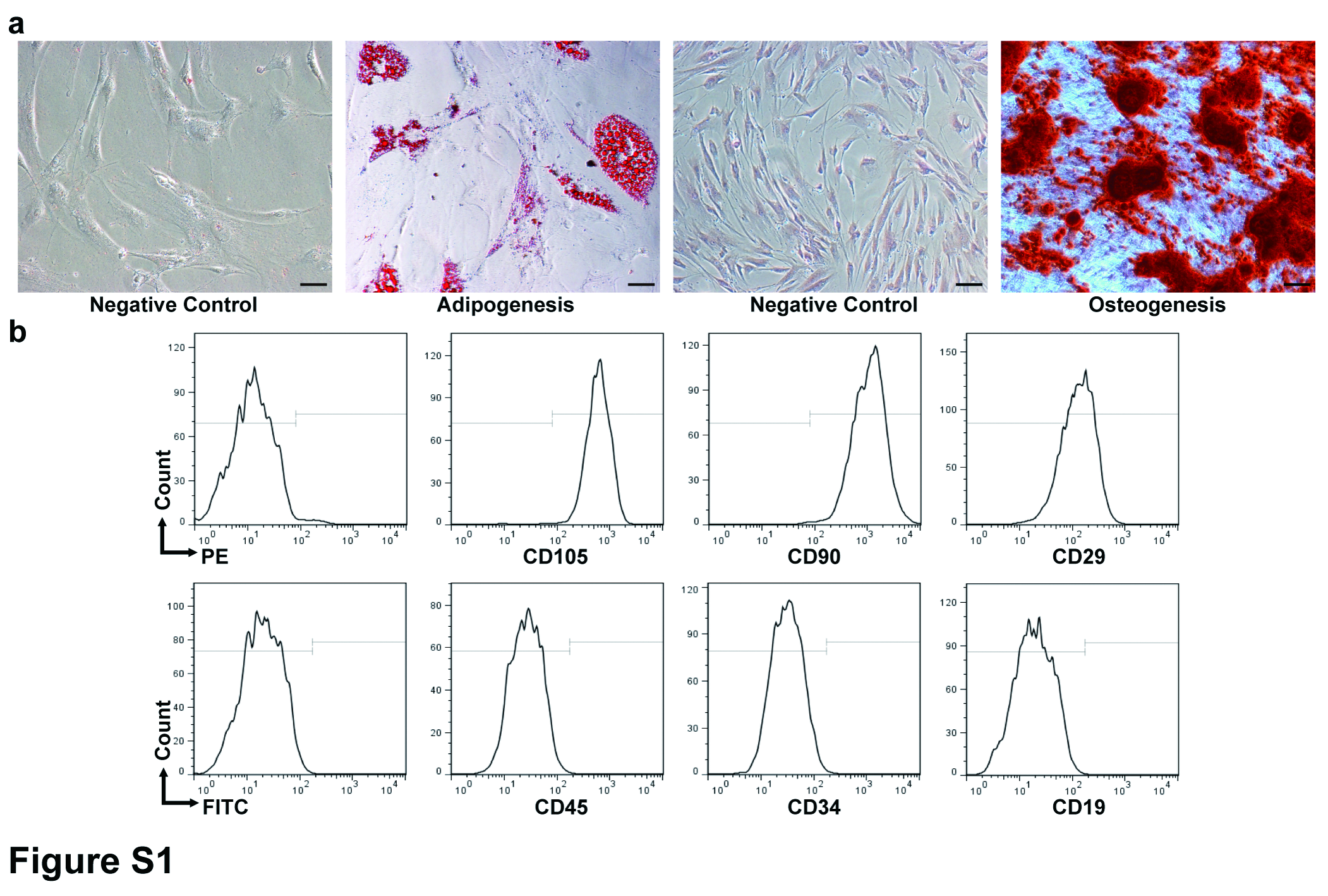

Supplement: Supplementary file 1 — Figure S1 [file 41419_2018_988_MOESM1_ESM.tif]

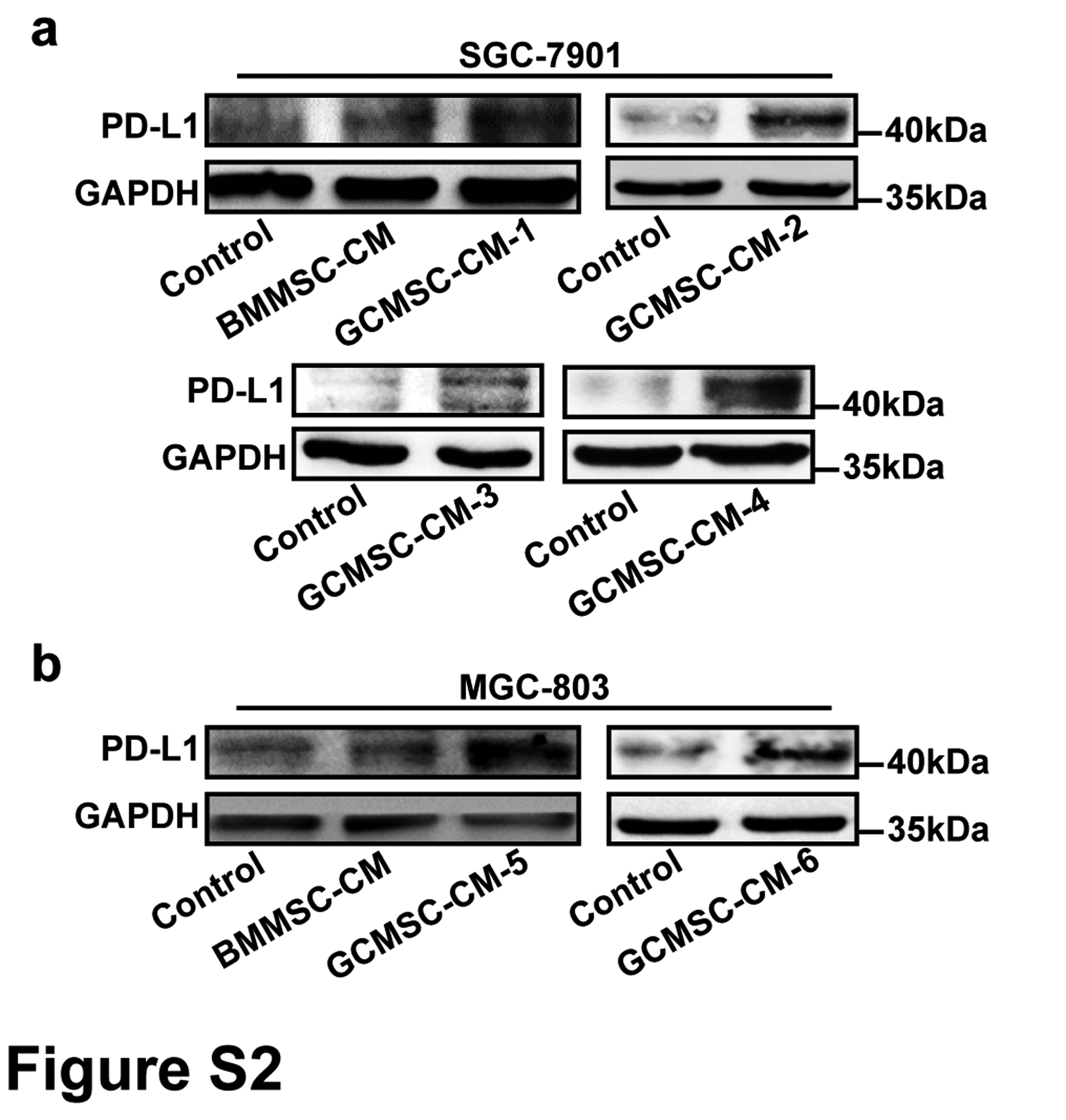

Supplement: Supplementary file 2 — Figure S2 [file 41419_2018_988_MOESM2_ESM.tif]

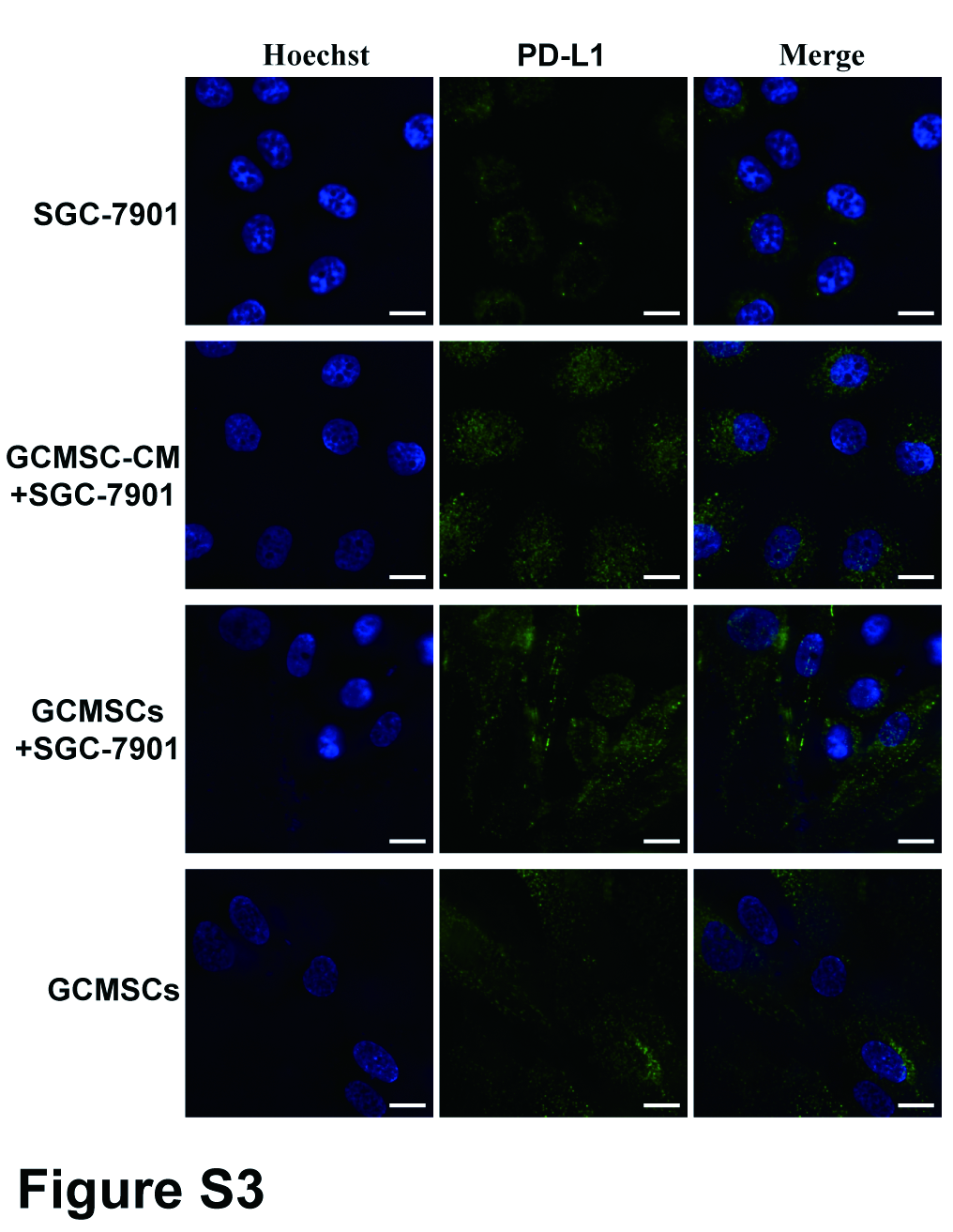

Supplement: Supplementary file 3 — Figure S3 [file 41419_2018_988_MOESM3_ESM.tif]

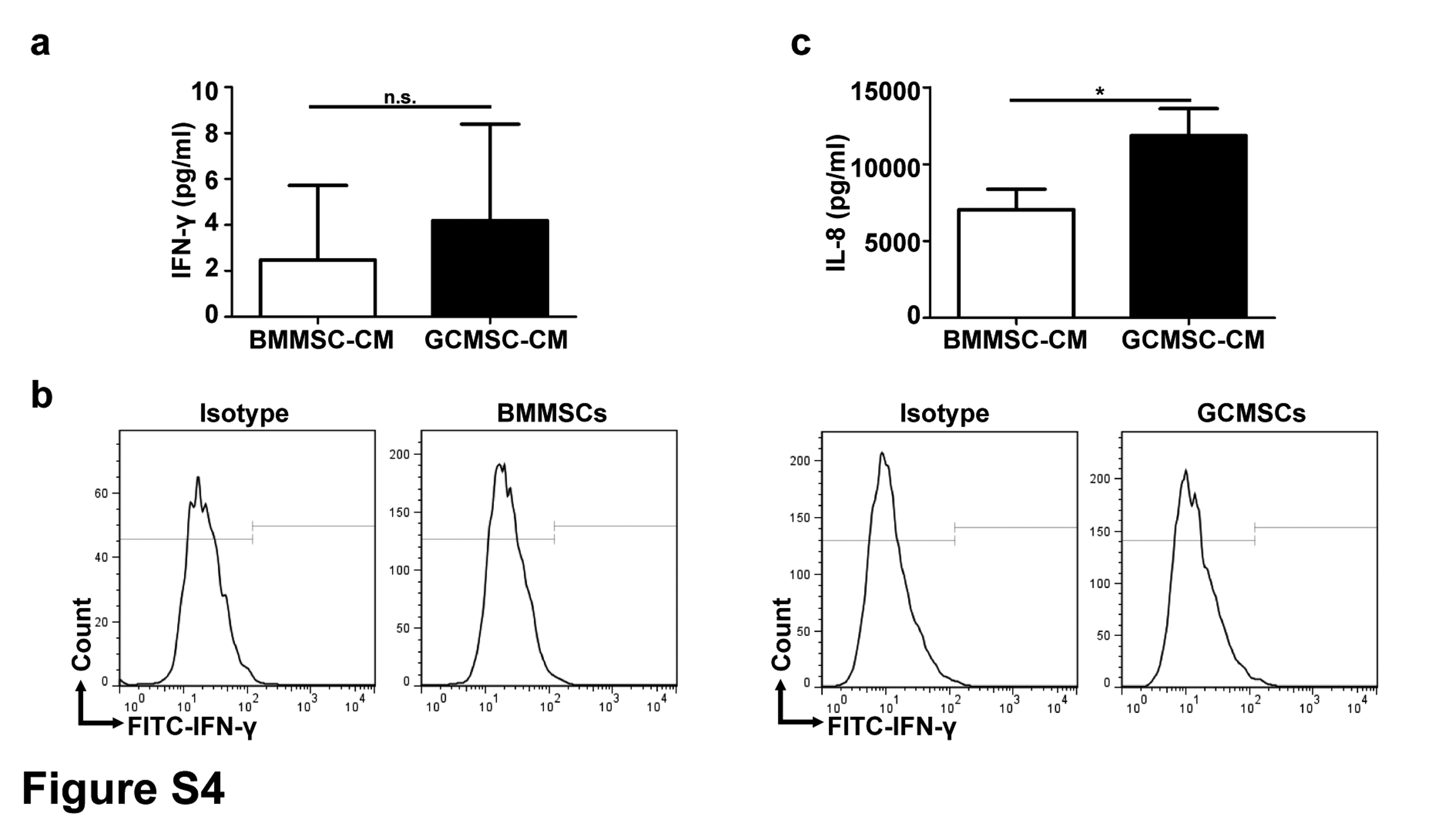

Supplement: Supplementary file 4 — Figure S4 [file 41419_2018_988_MOESM4_ESM.tif]

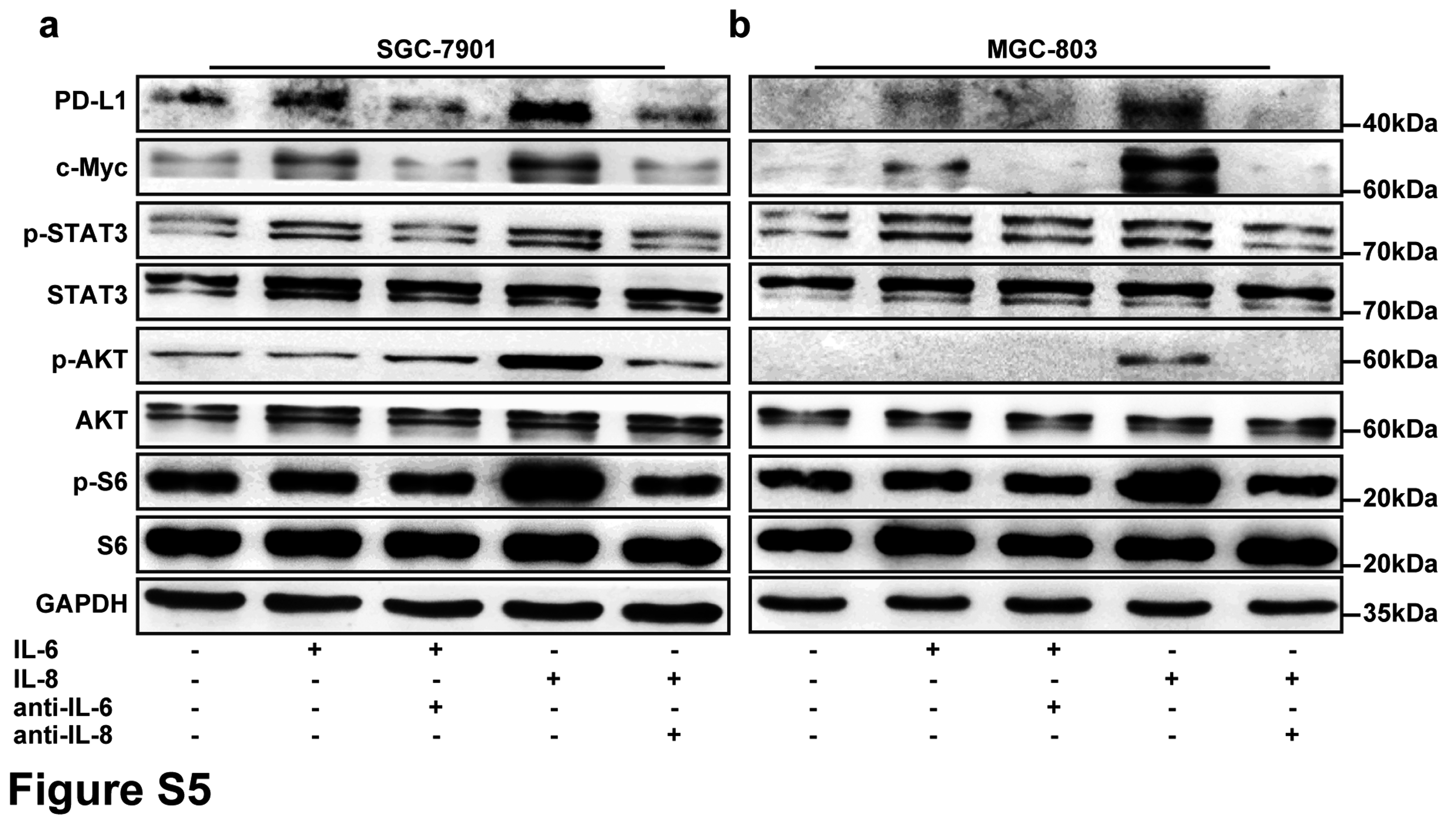

Supplement: Supplementary file 5 — Figure S5 [file 41419_2018_988_MOESM5_ESM.tif]
