# Supplementary material for: Gastric cancer mesenchymal stem cells derived IL-8 induces PD-L1 expression in gastric cancer cells via STAT3/mTOR-c-Myc signal axis
Source: Cell Death Dis. 2018 Sep 11;9(9):928. doi: 10.1038/s41419-018-0988-9 (PMC6134105; doi:10.1038/s41419-018-0988-9)
Supplement: Supplementary file 6 — supplementary figure legends [file 41419_2018_988_MOESM6_ESM.docx]

**Supplementary figure legends**

**Figure S1 Characterization of GCMSCs.** **a** Adipogenic differentiation (Bar = 50 μm) and Osteogenic differetiantion (Bar = 100 μm). **b** Flow cytometry analysis of the surface markers on GCMSCs.

**Figure S2 GCMSC-CM from different GC patients enhanced PD-L1 expression in GC cells.** GCMSC-CM used to treat **a** SGC-7901 and **b** MGC-803 was from a total of six different GC patients.

**Figure S3 The up-regulation of PD-L1 in GC cells mediated by GCMSCs was mainly via secretory cytokines.** SGC-7901 was co-cultured with GCMSCs or treated with GCMSC-CM for 24 h and the expression of PD-L1 was detected by [Immunofluorescence](javascript:;). Bar = 10 μm.

**Figure S4 The levels of INF-γ and IL-8 derived from GCMSCs. a** The secretion and **b** the expression of INF-γ by GCMSCs and BMMSCs. **c** The levels of IL-8 in GCMSC-CM and BMMSC-CM. Data in a and c represents the mean ± SD of three repeated experiments (n=3). BMMSCs and GCMSCs were isolated from three different healthy donors and GC patients, respectively. **P*<0.05, n.s., not significant.

**Figure S5 rhIL-8 induced PD-L1 expression in GC cells via c-Myc regulated by STAT3 and mTOR signaling pathways.** The expression of PD-L1 and the activation of signaling pathways in **a** SGC-7901 and **b** MGC-803 treated with rhIL-6 (15 ng/ml, Peprotech), rhIL-6+anti-IL-6, rhIL-8 (15 ng/ml, Peprotech) and rhIL-8+anti-IL-8, respectively.
